# Multilocus genetic analyses and spatial modeling reveal complex population structure and history in a widespread resident North American passerine (*Perisoreus canadensis*)

**DOI:** 10.1002/ece3.3478

**Published:** 2017-10-20

**Authors:** Kimberly M. Dohms, Brendan A. Graham, Theresa M. Burg

**Affiliations:** ^1^ Department of Biological Sciences University of Lethbridge Lethbridge AB Canada; ^2^ Department of Biological Sciences University of Windsor Windsor ON Canada; ^3^ Present address: Canadian Wildlife Service, Environment and Climate Change Canada Delta BC Canada

**Keywords:** barriers, corvid, gene flow, *Perisoreus canadensis*, Pleistocene, refugia

## Abstract

An increasing body of studies of widely distributed, high latitude species shows a variety of refugial locations and population genetic patterns. We examined the effects of glaciations and dispersal barriers on the population genetic patterns of a widely distributed, high latitude, resident corvid, the gray jay (*Perisoreus canadensis*), using the highly variable mitochondrial DNA (mtDNA) control region and microsatellite markers combined with species distribution modeling. We sequenced 914 bp of mtDNA control region for 375 individuals from 37 populations and screened seven loci for 402 individuals from 27 populations across the gray jay range. We used species distribution modeling and a range of phylogeographic analyses (haplotype diversity, Φ_ST_, SAMOVA,* F*_ST_, Bayesian clustering analyses) to examine evolutionary history and population genetic structure. MtDNA and microsatellite markers revealed significant genetic differentiation among populations with high concordance between markers. Paleodistribution models supported at least five potential areas of suitable gray jay habitat during the last glacial maximum and revealed distributions similar to the gray jay's contemporary during the last interglacial. Colonization from and prolonged isolation in multiple refugia is evident. Historical climatic fluctuations, the presence of multiple dispersal barriers, and highly restricted gene flow appear to be responsible for strong genetic diversification and differentiation in gray jays.

## 1 INTRODUCTION

During the last glacial maximum (LGM), large portions of North America were covered by ice sheets (Pielou, 1991), fragmenting species’ ranges, and restricting surviving individuals and populations to ice‐free refugia. Long‐term isolation in glacial refugia has been shown to promote genetic diversification in a variety of organisms (Jaramillo‐Correa, Beaulieu, Khasa, & Bousquet, 2009; Shafer, Cullingham, Côté, & Coltman, 2010; Weir & Schluter, 2004). North American plant and animal species expanded from several known refugia following the retreat of the ice sheets, including Beringia (parts of Alaska) and three areas south of the ice sheets (Pacific Coast, Rockies, and Taiga), while coastal areas such as Newfoundland are contested to have been ice‐free (Jaramillo‐Correa et al., 2009; Pielou, 1991). Contemporary genetic patterns are strongly influenced by postglacial expansion from refugia (Weir & Schluter, 2004; Williams, 2003), historical and contemporary barriers to dispersal (Brunsfeld, Sullivan, Soltis, & Soltis, 2001; Keyghobadi, 2007; Schwalm, Waits, & Ballard, 2014), and dispersal potential (Burg, Lomax, Almond, Brooke, & Amox, 2003; Riginos, Buckley, Blomberg, & Treml, 2014).

Historical events shaping current population structure should be particularly evident in resident species. Sedentary species generally retain patterns of genetic variation longer due to limited dispersal, allowing researchers to make inferences about past historic events (Burg, Gaston, Winker, & Friesen, 2005, 2006; Jaramillo‐Correa et al., 2009; Petit et al., 2005). Tree species, for example, show distinct patterns of population genetic structure and the influence of historical environmental changes (Jaramillo‐Correa et al., 2009; Morris, Graham, Soltis, & Soltis, 2010; Roberts & Hamann, 2015). Similar patterns are emerging in vertebrate taxa as the number of studies on resident species increases (e.g., Adams & Burg, 2015; Arbogast, Browne, & Weigl, 2001; Barrowclough, Groth, Mertz, & Gutiérrez, 2004; Burg et al., 2005; Graham & Burg, 2012).

**Figure 1:**
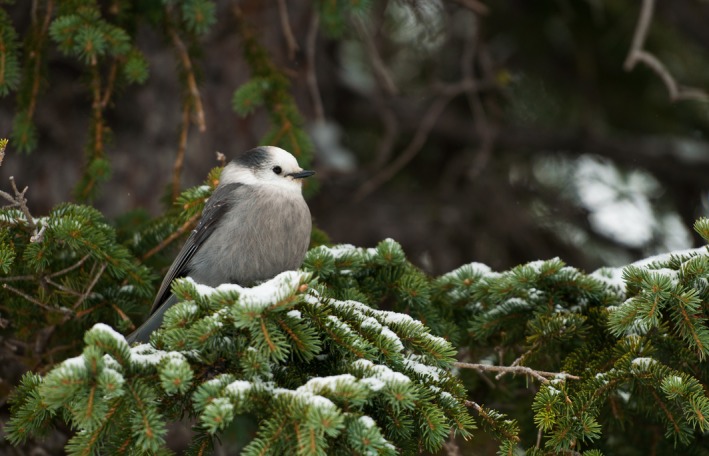
Gray jay (*Perisoreus canadensis*) in the boreal forest of Waterton Lakes National Park, Alberta, Canada. Copyright: Kimberly Dohms (2012)

**Figure 2:**
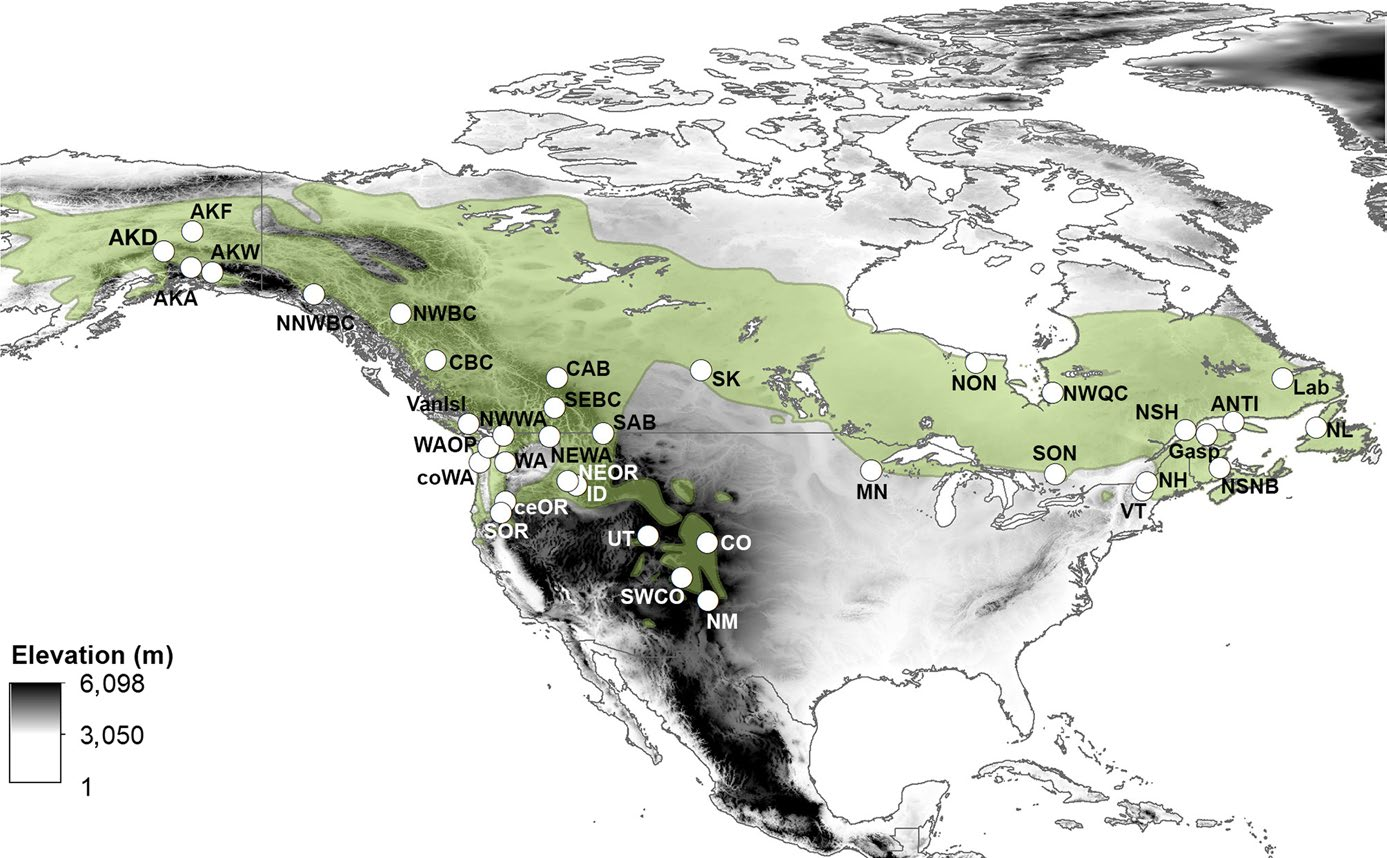
Sampled gray jay populations. Gray jay range (light green) in North America and central location of sampled populations (white circles) overlaid on digital elevation model of North America. Population abbreviations and locations are given in Table 1

The gray jay (*Perisoreus canadensis*; Figure 1) is ideal for investigating patterns of postglacial colonization and the impact of dispersal barriers on resident species for several reasons. Gray jays are a relatively sedentary species, like their putative sister species the Siberian jay (*Perisoreus infaustus*; Strickland & Ouellet, 2011), which exhibits strong population genetic structure in fragmented habitats (Uimaniemi et al., 2000). Adult gray jays remain in the same territory between breeding seasons, and natal dispersal is limited to nearby territories, though some irruptive juvenile dispersal has been observed (Strickland & Ouellet, 2011). Gray jays are broadly distributed across northern and western North America (Figure 2) and strongly associated with spruce (*Picea* spp.). Gray jay contemporary range encompasses a number of purported barriers to dispersal (e.g., Salish Sea, Strait of Belle Isle, Columbia Basin), in addition to previously glaciated (e.g., most of Canada) and unglaciated areas (e.g., Alaska, western United States). Gray jays display plumage and morphological trait variation across their range (Strickland & Ouellet, 2011). The presence of distinct morphs suggests the potential for reduced gene flow and population structure (Arnoux et al., 2014; Burg et al., 2005; Miller‐Butterworth, Jacobs, & Harley, 2003), though morphological characteristics have also been shown to vary with temperature and other environmental variables (Diniz‐Filho et al., 2009).

Using both mitochondrial DNA and nuclear microsatellite markers, we examine genetic structure and the effect of Pleistocene glaciations and dispersal barriers on genetic variation in this species. A previous study by van Els, Cicero, and Klicka (2012) using mtDNA data found that gray jays exhibit high levels of genetic diversity and genetic structure throughout their range; these patterns likely stem from populations residing in multiple ice‐free refugia during the LGM. Although this study had a relatively large sample size (*n* = 205), many of the sites included in the study had small sample sizes (mean = 3.9 individuals/site). Here, we use expanded sampling to include more populations from previously glaciated areas and incorporate more sites from the full distribution of gray jays. In addition, incorporating both mtDNA and microsatellite markers allows us to compare historical (mtDNA) and contemporary (microsatellite) genetic patterns in this species. Based on limited dispersal, patterns of glaciation during the LGM, and present distribution, we predict that gray jays expanded from multiple refugia throughout North America, and will exhibit high levels of genetic divergence between populations separated by barriers to dispersal.

## 2 MATERIALS AND METHODS

### 2.1 Sample collection

From 2007 to 2012, we captured gray jays at each sampling site (hereafter referred to as a population) using standard mistnetting techniques with call playback. We limited mistnetting locations to within a 50 km radius and sites contained no obvious barriers to dispersal. Sampling sites were paired in two ways: (1) located in areas that were previously glaciated and unglaciated during the last glacial maximum and (2) on either side of possible barriers to dispersal (Figure 2). We collected less than 100 μl of blood from each bird, and blood was stored in 95% ethanol. Each bird was banded with a US Fish & Wildlife Service aluminum band, and aged and sexed when possible using standard procedures and protocols (Tables S1–S5). Additional genetic samples were obtained from museum collections taken from birds during the breeding season within the past 20 years (Table 1; Table S1). DNA was extracted from blood, tissue, and feather samples using a modified Chelex protocol (Burg & Croxall, 2001; Walsh, Metzger, & Higuchi, 1991).

**Table 1:** Summary table of gray jay samples and mitochondrial DNA information from analyses . Italicized values are overall for corresponding genetic group

Genetic Group	Pop	Lat (*N*)	Long (W)	*n*	*H*_n_	*H*_d_	π
Boreal‐east				*203*	*163*	*0.998*	*0.008*
	AKA	62.12	−146.57	8	8	1.000	0.012
	AKF	64.95	−146.47	8	7	0.936	0.010
	AKW	61.71	−144.88	17	14	0.969	0.007
	AKD	63.38	−148.47	1	1	–	–
	NWBC	58.45	−130.00	15	11	0.952	0.008
	NNWBC	60.00	−136.87	9	5	0.707	0.004
	CBC	54.77	−127.27	13	10	0.949	0.010
	CAB	53.39	−117.68	20	15	0.968	0.010
	SK	53.97	−106.29	11	9	0.913	0.010
	MN	46.13	−92.87	3	2	0.728	–
	NON	54.56	−84.63	14	9	0.973	0.004
	NWQC	52.24	−78.56	11	11	1.000	0.005
	SON	45.80	−78.56	16	16	1.000	0.005
	Gasp	48.93	−66.40	2	2	1.000	–
	NSH	49.27	−68.09	2	2	1.000	–
	ANTI	49.27	−64.31	11	7	0.728	0.003
	NSNB	46.30	−65.38	6	4	0.800	0.006
	VT	44.55	−71.47	20	13	0.852	0.007
	NH	45.18	−71.15	3	2	0.925	–
	Lab	53.34	−60.41	17	15	0.979	0.005
NL	NL	49.46	−57.76	*12*	*8*	0.897	0.002
UT	UT	40.57	−110.47	*12*	*7*	0.897	0.003
IMW				*40*	*37*	*0.996*	*0.009*
	SAB	49.04	−114.03	13	13	1.000	0.007
	NEWA	48.76	−118.25	11	9	0.913	0.014
	NEOR	45.26	−116.84	10	8	0.955	0.006
	ID	44.95	−116.14	3	3	1.000	–
	SEBC	51.04	−117.87	3	3	1.000	–
CO–NM				*37*	*30*	*0.993*	*0.005*
	CO	40.41	−105.82	20	15	0.949	0.005
	SWCO	37.63	−107.83	12	12	1.000	0.009
	NM	35.81	−105.79	5	5	1.000	0.002
Pacific Coast				*52*	*37*	*0.957*	*0.004*
	WA	46.77	−121.75	33	19	0.938	0.004
	coWA	46.74	−123.80	6	4	0.903	0.002
	NWWA	48.89	−121.90	4	3	0.823	0.003
	WAOP	47.94	−123.07	3	3	1.000	–
	ceOR	43.65	−121.76	5	4	0.900	0.004
	SOR	42.78	−122.08	1	1	–	–
VanIsl	VanIsl	49.74	−124.68	16	10	0.975	0.002
**Overall**				**375**	**261**	**0.982**	**0.061**

Latitude and longitude are central points for population sampling sites. *H*_d_, mitochondrial DNA haplotype and π, nucleotide diversity (multiplied by 100 for ease of viewing). See Table S1 for additional museum collection information including voucher/specimen numbers, latitude and longitude, and sex.

### 2.2 Laboratory procedures

#### 2.2.1 Mitochondrial DNA

We amplified a section of the mitochondrial DNA control region (CR) using primers L46 SJ (5′‐TTT GGC TAT GTA TTT CTT TGC‐3′; Birt & Lemmen, unpublished data) and H1030 JCR 18 (5′‐TAA ATG ATT TGG ACA ATC TAG G‐3′; Saunders & Edwards, 2000), corresponding to position 46 (Domain I) to 1030 (Domain III) of the corvid mitochondrial control region. Where the complete fragment would not amplify, we used internal primers designed in‐house, H590 grjaCR (5′‐GGA GTA TGC ATC CGA CCA CT‐3′) with L46 SJ or L530 corvidae (5′‐CGC CTC TGG TTC CTA TTT CA‐3′) with H1030 JCR 18, to amplify two overlapping fragments. PCRs were performed on a Master gradient thermocycler (Eppendorf: Hauppauge, NY) in 25 μl reactions with 1× goTaq Flexi buffer (Promega: Madison, WI, USA), 2.5 mmol/L MgCl_2_, 200 μmol/L dNTP, 0.4 μmol/L of each primer, and 0.5 units goTaq Flexi taq polymerase (Promega) under the following conditions: one cycle of 94°C for 120 s, 52°C for 45 s, and 72°C for 60 s, 37 cycles of 94°C for 30 s, 52°C for 45 s and 72°C for 60 s and one cycle of 72°C for five min. PCR products were run on a 0.8% agarose gel to confirm DNA amplification.

DNA sequencing was performed at McGill University and Génome Québec Innovation Centre on a 3730xl DNA Analyzer (Applied Biosystems: Carlsbad, CA, USA) or at the University of Lethbridge on a 3130 DNA Analyzer (Applied Biosystems). For in‐house sequencing, we used a shrimp alkaline phosphatase‐exonuclease clean up followed by sequencing and sodium acetate precipitation (Graham & Burg, 2012) before electrophoresis.

#### 2.2.2 Microsatellite DNA

We screened a subset of individuals at 30 microsatellite primer pairs developed for and used in other corvids. Seven of the 30 loci were polymorphic. To allow for integration of a fluorescently labeled primer (700 or 800 nm) directly into the PCR product, we modified all forward primers by adding an M13 sequence (5′‐CAC GAC GTT GTA AAA CGA C‐3′) to the 5′ end. DNA was amplified in a 10 μl reaction with 1× buffer, 1 mmol/L MgCl_2_, 200 μmol/L dNTP (Fisher Scientific), 1 μmol/L of each primer (forward and reverse), 0.05 μmol/L of the fluorescent primer (Eurofins MWG Operon) and 0.5 units taq polymerase under the following conditions: one cycle of 94°C for 120 s, T_1_ for 45 s, and 72°C for 60 s, seven cycles of 94°C for 60 s, T_1_ for 30 s and 72°C for 45 s, 31 cycles of 94°C for 30 s, T_2_ for 30 s, and 72°C for 45 s, and one final elongation cycle at 72°C for 5 min (Table S2). PCR products were mixed with a stop solution (95% formamide, 20 mmol/L EDTA and bromophenol blue), denatured for 3 min at 94°C, then run on a 6% polyacrylamide gel using a LI‐COR 4300 DNA Analyzer (LI‐COR Inc., Lincoln, NE). Alleles were scored via visual inspection, and genotypes were independently confirmed by a second person. Three controls of known allele sizes (pre‐screened individuals) plus a size standard were included on each load to ensure consistent scoring along with a negative control to ensure no contamination was present.

### 2.3 Analyses of genetic structure

#### 2.3.1 Mitochondrial DNA

We edited and aligned sequences from chromatograms using mega v 5.0 (Tamura et al., 2011). To assess population structure and evaluate relationships among haplotypes, we constructed a statistical parsimony network (95% probability) using tcs v 1.21 (Clement, Posada, & Crandall, 2000). We measured genetic variation within populations and haplogroups by calculating haplotype (H_d_) and nucleotide (π) diversity using arlequin v 3.11 (Excoffier, Laval, & Schneider, 2005). To examine population structure and assess genetic differentiation among populations and haplogroups, we calculated pairwise Φ_ST_ values (an analogue of Wright's fixation index *F*_ST_) using arlequin v 3.11 (Excoffier et al., 2005). We corrected significance values using a Benjamini–Hochberg correction (Benjamini & Hochberg, 1995) to control for false discovery rate (FDR). We examined genetic structure within and among populations by performing an analysis of molecular variance (AMOVA) in arlequin v 3.11 (Excoffier et al., 2005) and used a spatial analysis of molecular variance (SAMOVA; Dupanloup, Schneider, & Excoffier, 2002) approach to assess barriers between gray jay populations.

To reconstruct the phylogenetic relationship among populations, we used the Bayesian inference program MrBayes 3.2 (Ronquist et al., 2012). For our analyses, we analyzed all CR haplotypes using a GTR G+I model as this was the best‐fit model, as determined in JModelTest (version 0.1.1; Posada, 2008). We ran the analyses for 10 million generations using four chains, sampling every 100th generation. We used a burn‐in percentage of 25%, using the remaining trees to construct consensus trees, which we viewed using FIGTREE 1.3.1 (Rambaut & Drummond, 2006).

#### 2.3.2 Microsatellite DNA

Allelic richness was calculated in fstat v2.9.3 (Goudet, 2001). Allele frequencies, observed (*H*_o_) and expected (*H*_e_) heterozygosities, and pairwise *F*_ST_ values (Wright, 1978) were calculated with 1000 permutations using arlequin v 3.11 (Excoffier et al., 2005). We corrected *p* values for multiple tests using a Benjamini–Hochberg correction (Benjamini & Hochberg, 1995) to control for FDR.

Bayesian clustering analyses were conducted using Structure v2.3.3 (Falush, Stephens, & Pritchard, 2003; Pritchard, Stephens, & Donnelly, 2000); we used the following settings for our initial run examining all 27 populations: a burn‐in of 100,000 followed by 500,000 runs, admixture assumed, correlated allele frequencies without population information as an a priori. Ten replicates were performed for each value of *K*. In structure, it can be difficult to decide when *K* captures major structure in the data due to similar lnP(X|K) values, thus structure harvester (Earl & von Holdt, 2012) was used to confirm the most parsimonious clustering of groups. Following our initial run that included all 27 populations, we tested for hierarchical structure, following the procedure used by Adams and Burg (2015). For these runs, we used the same settings as our initial run, although we used a burn‐in of 50,000 followed by 100,000 chains.

### 2.4 Species distribution and paleodistribution modeling

We used species distribution modeling (SDM) to construct a model of current, LGM (~21 ka), and Last Interglacial (LIG; ~120–140 ka) gray jay distributions. Geo‐referenced locations were obtained from the Global Biodiversity Information Facility (GBIF; http://data.gbif.org/, accessed on 3 October 2011). Data were inspected and occurrences outside of North America, without geo‐referencing, or recorded before 1950 were excluded from the analyses. From the GBIF data, we trained and tested the models using location records from field data, multiple museums, Animal Sound Archive Berlin, Borror Laboratory of Bioacoustics, Macaulay Library Audio Data, USDA Forest Service Lamna Point Count, Point Reyes Bird Observatory Point Counts, Ontario Breeding Bird Atlas 1981–1985 and 2001–2005, and Northwest Territories and Nunavut Bird Checklist. Duplicate records and remaining outliers were removed prior to model‐building.

We extracted current bioclimatic data from the WORLDCLIM dataset (v 1.4, http://www.worldclim.org/) at 2.5 min and 30 arc‐seconds resolution, LGM bioclimatic data from the Model for Interdisciplinary Research on Climate (MIROC) dataset at 2.5‐min resolution (Hasumi & Emori, 2004), and LIG bioclimatic data from Otto‐Bliesner, Marshall, Overpeck, and Miller (2006) at 30 arc‐seconds resolution. The current bioclimatic dataset ranges over a 50‐year period (1950–2000), hence we excluded gray jay observations prior to 1950 for consistency. Nineteen bioclimatic variables are included in the WORLDCLIM current and LGM (Hijmans, Cameron, Parra, Jones, & Jarvis, 2005) and LIG (Otto‐Bliesner et al., 2006) datasets. We used ArcGIS 9.3 (ESRI: Redlands, CA) to clip climatic variable layers to include only North America as using smaller geographic areas can improve predictive power of maxent models (Anderson & Raza, 2010). Prior to constructing SDM, we used ENMtools (v 1.3; Warren, Glor, & Turelli, 2010) to determine which bioclimatic variables were correlated, using *R* > 0.90 as a cutoff. Nine variables were correlated with at least one other variable, and all but one from each set of correlated variables were removed.

maxent (v 3.3.3; Phillips, Anderson, & Schapire, 2006) was used to model current and past gray jay distribution. We used the following settings for the maxent model: hinge features only, regularization multiplier of 1, 10,000 max number of background points, replicate run type of 10 cross‐validations, 500 maximum iterations, and 0.00001 convergence threshold. We used hinge features only as these are appropriate for samples of greater than 15, improve model performance, and allow for simpler approximations of species response to the environment (Phillips & Dudik, 2008). We ran jackknife tests to measure the importance of each bioclimatic variable. Models used 1,447 range‐wide presence records for training, 161 records for testing and 10 BIOCLIM environmental layers (bio1‐4, 8, 12, 14‐15, 18‐19) to produce models for present and paleodistributions.

### 2.5 Correlates predicting genetic structure

We used two separate approaches to examine the factors that influence genetic structure. First we used the program BARRIER to identify potential barriers that may contribute to genetic structure. BARRIER uses Delaunay triangulation and Monmonier's distance matrix to identify potential barriers. We identified the first 10 genetic barriers using both our mtDNA and microsatellite datasets; distance matrices were created using pairwise Φ_ST_ and *F*_ST_ values. We identified barriers with each dataset separately, so that we could compare patterns between markers and determine if similar barriers influence historical and contemporary genetic patterns.

Next, we used a distance‐based redundancy analysis (dbRDA) to test the role of ecological variables on genetic variation. We ran two separate analyses, one for mtDNA genetic variation and a second for microsatellite genetic variation. DbRDA is a multivariate approach to test the effect of multiple predictor variables on one or more response variables (Legendre & Legendre, 1998). Although Mantel tests are often used to measure the relationship between genetic matrices and other distance matrices, recent studies have suggested that canonical statistical approaches like dbRDA are better suited for examining questions where distance matrices are not applicable (Legendre & Fortin, 2010). This approach is especially useful for studies examining the influence of environmental variation or other abiotic factors because it allows for the testing of those variables directly.

To construct our dbRDA models, we used the “capscale” function in the R package Vegan (R Core Team, 2016). We performed this analysis at the individual level so that we could examine the full‐extent genetic variation in both mtDNA and microsatellite patterns. For our response variable, we calculated Nei's genetic distance between all individuals for mtDNA and microsatellite datasets using GenAlEx (Peakall & Smouse, 2006). We examined six predictor variables in our models, including geographic location (latitude and longitude) for each individual and geographic distance. For our geographic distance, we used the first principal coordinate for each individual; similar to our genetic response variables, we performed a principal coordinate analysis in GenAlEx on a geographic distance matrix following the approach of Kierepka & Latch, (2016). For our remaining four variables, we used information obtained from our spatial distribution models. We examined the influence of mean annual temperature and precipitation during the coldest quarter, as these were the two most important variables that predicted gray jay distributions in those models. Additionally, we examined the role of altitude, which we obtained from the BIOCLIM dataset. All three variables were obtained using “the point sampling” tool in QGIS (Quantum GIS Team, 2017). Finally, we examined the effect of glaciation by scoring an area as glaciated or unglaciated based on the results of our spatial distribution modeling results from the last interglacial.

## 3 RESULTS

### 3.1 Genetic structure

We collected samples from and genotyped mitochondrial DNA of 375 individual gray jays from 37 populations (Table 1, Figure 2) and seven polymorphic microsatellite loci for 402 individuals from the 27 populations with five or more samples from across the range (Table 2).

**Table 2:** Summary table of seven microsatellite loci used to analyze gray jay populations

	ApCo30	ApCo37	ApCo40	ApCo41	ApCo91	Ck2A5A	MJG1
AKA (*n* = 8)							
*A*_n_	5	5	6	2	3	2	1
*A*_r_	3.47	3.26	4.04	4.04	4.04	4.04	1.00
*H*_o_	0.86	0.75	0.67	0.63	0.500	0.13	0.00
*H*_e_	0.70	0.66	0.75	0.43	0.40	0.12	0.00
P	ns	ns	ns	ns	ns	ns	–
AKF (*n* = 8)							
*A*_n_	4	6	6	1	5	2	1
*A*_r_	3.12	3.13	4.26	4.26	4.26	4.26	1.00
*H*_o_	0.57	0.50	0.80	0.00	0.14	0.40	0.00
*H*_e_	0.65	0.58	0.76	0.00	0.72	0.48	0.00
P	ns	ns	ns	–	*	ns	–
AKW (*n* = 18)							
*A*_n_	5	4	5	2	4	1	1
*A*_r_	2.83	2.86	2.97	2.97	2.97	2.97	1.00
*H*_o_	0.44	0.69	0.31	0.11	0.27	0.00	0.00
*H*_e_	0.56	0.64	0.63	0.11	0.48	0.00	0.00
P	ns	ns	ns	ns	**	–	–
NWBC (*n* = 16)							
*A*_n_	4	9	6	1	4	1	1
*A*_r_	3.29	3.69	3.82	3.82	3.82	3.82	1.00
*H*_o_	0.79	0.69	1.00	0.00	0.62	0.00	0.00
*H*_e_	0.72	0.71	0.77	0.00	0.64	0.00	0.00
P	*	*	**	–	ns	–	–
NNWBC (*n* = 9)							
*A*_n_	4	6	6	1	3	1	1.00
*A*_r_	2.63	3.69	4.50	4.50	4.50	4.50	1.00
*H*_o_	0.44	0.38	0.80	0.00	0.43	0.00	0.00
*H*_e_	0.51	0.73	0.80	0.00	0.36	0.00	0.00
P	ns	*	ns		ns		
CBC (*n* = 13)							
*A*_n_	3	4	5	2	6	3	2.00
*A*_r_	2.78	2.51	3.72	3.72	3.72	3.72	1.42
*H*_o_	0.50	0.15	0.91	0.08	0.46	0.55	0.15
*H*_e_	0.65	0.49	0.77	0.07	0.68	0.53	0.14
P	ns	***	ns	ns	ns	ns	ns
CAB (*n* = 28)							
*A*_n_	6	5	8	1	5	3	2
*A*_r_	3.33	2.15	3.90	3.90	3.90	3.90	1.81
*H*_o_	0.78	0.38	0.71	0.00	0.50	0.23	0.00
*H*_e_	0.69	0.37	0.79	0.00	0.42	0.21	0.35
P	ns	ns	*		ns	ns	***
SK (*n* = 11)							
*A*_n_	5	4	9	2	3	2	2
*A*_r_	3.57	2.52	4.55	4.55	4.55	4.55	1.48
*H*_o_	0.82	0.73	0.89	0.20	0.38	0.11	0.18
*H*_e_	0.75	0.58	0.83	0.18	0.32	0.11	0.17
P	ns	ns	ns	ns	ns	ns	ns
NON (*n* = 26)							
*A*_n_	7	7	10	3	5	5	3
*A*_r_	3.68	2.89	4.31	4.31	4.31	4.31	1.43
*H*_o_	0.54	0.27	0.85	0.08	0.52	0.33	0.12
*H*_e_	0.76	0.62	0.84	0.08	0.62	0.30	0.14
P	*	***	ns	ns	*	ns	***
NWQC (*n* = 11)							
*A*_n_	6	3	7	3	2	2	2
*A*_r_	3.82	2.23	4.06	4.06	4.06	4.06	1.27
*H*_o_	0.56	0.46	0.60	0.27	0.14	0.09	0.09
*H*_e_	0.77	0.52	0.79	0.42	0.13	0.09	0.09
P	*	ns	ns	ns	ns	ns	ns
SON (*n* = 17)							
*A*_n_	5	5	9	3	1	2	1
*A*_r_	3.49	2.91	4.34	4.34	4.34	4.34	1.00
*H*_o_	0.43	0.33	0.87	0.25	0.00	0.07	0.00
*H*_e_	0.71	0.64	0.83	0.37	0.00	0.06	0.00
P	ns	ns	*	***		ns	
*A*_n_ TI (*n* = 12)							
*A*_n_	2	2	5	1	3	2	1
*A*_r_	1.89	1.99	3.48	3.48	3.48	3.48	1.00
*H*_o_	0.36	0.64	1.00	0.00	0.58	0.08	0.00
*H*_e_	0.40	0.50	0.74	0.00	0.52	0.22	0.00
P	ns	ns	ns		ns	*	
NSNB (*n* = 5)^a^							
*A*_n_	3	3	4	1	3	1	2
*A*_r_	2.47	2.20	4.00	4.00	4.00	4.00	2.00
*H*_o_	0.60	0.40	1.00	0.00	0.50	0.00	0.33
*H*_e_	0.46	0.34	0.72	0.00	0.53	0.00	0.28
P	ns	ns	ns		ns		ns
VT (*n* = 39)							
*A*_n_	7	6	8	3	4	3	3
*A*_r_	3.32	2.50	3.67	3.67	3.67	3.67	1.45
*H*_o_	0.74	0.46	0.77	0.11	0.32	0.32	0.10
*H*_e_	0.70	0.48	0.77	0.11	0.41	0.31	0.16
P	ns	ns	*	ns	***	ns	ns
Lab (*n* = 18)							
*A*_n_	3	5	9	2	4	6	2
*A*_r_	2.51	2.34	4.58	4.58	4.58	4.58	1.21
*H*_o_	0.24	0.44	0.63	0.19	0.46	0.47	0.07
*H*_e_	0.56	0.45	0.86	0.17	0.52	0.48	0.07
P	**	ns	**	ns	ns	**	ns
NL (*n* = 12)							
*A*_n_	4	3	9	3	3	2	1
*A*_r_	3.40	1.75	4.71	4.71	4.71	4.71	1.00
*H*_o_	0.42	0.27	0.82	0.20	0.67	0.33	0.00
*H*_e_	0.74	0.24	0.86	0.19	0.49	0.28	0.00
P	*	ns	ns	ns	ns	ns	
UT (*n* = 12)							
*A*_n_	2	6	5	2	4	1	1
*A*_r_	1.27	3.39	3.51	3.51	3.51	3.51	1.00
*H*_o_	0.09	0.75	0.56	0.17	0.56	0.00	0.00
*H*_e_	0.09	0.70	0.72	0.15	0.69	0.00	0.00
P	ns	ns	*	ns	ns		
SAB (*n* = 13)							
*A*_n_	6	5	6	2	3	1	1
*A*_r_	3.58	2.39	4.67	4.67	4.67	4.67	1
*H*_o_	0.60	0.50	1.00	0.08	0.33	0.00	0.00
*H*_e_	0.72	0.42	0.82	0.07	0.49	0.00	0.00
P	ns	ns	ns	ns	ns		
NEWA (*n* = 12)							
*A*_n_	4	4	10	3	5	2	6
*A*_r_	3.13	2.38	4.68	4.68	4.68	4.68	2.71
*H*_o_	0.50	0.55	0.82	0.18	0.92	0.50	0.67
*H*_e_	0.69	0.48	0.86	0.17	0.68	0.38	0.52
P	ns	ns	*	ns	ns	ns	ns
NEOR (*n* = 11)							
*A*_n_	3	3	7	2	4	2	3
*A*_r_	2.02	2.57	4.23	4.23	4.23	4.23	1.90
*H*_o_	0.46	0.88	0.71	0.20	0.44	0.25	0.33
*H*_e_	0.37	0.57	0.79	0.32	0.38	0.22	0.29
P	ns	ns	ns	ns	ns	ns	ns
CO (*n* = 19)							
*A*_n_	5	4	5	2	6	5	2
*A*_r_	3.39	3.01	3.35	3.35	3.357	3.35	1.54
*H*_o_	0.37	0.50	0.67	0.16	0.71	0.47	0.00
*H*_e_	0.73	0.67	0.72	0.15	0.72	0.44	0.20
P	*	*	ns	ns	ns	ns	***
SWCO (*n* = 12)							
*A*_n_	3	4	6	1	3	1	1
*A*_r_	2.02	2.65	3.50	3.50	3.50	3.50	1.00
*H*_o_	0.09	0.50	0.75	0.00	0.83	0.00	0.00
*H*_e_	0.37	0.60	0.73	0.00	0.60	0.00	0.00
P	*	*	ns		ns		
NM (*n* = 5)							
*A*_n_	1	2	4	1	1	1	1
*A*_r_	1.00	1.87	3.43	3.43	3.43	3.43	1.00
*H*_o_	0.00	0.00	1.00	0.00	0.00	0.00	0.00
*H*_e_	0.00	0.32	0.70	0.00	0.00	0.00	0.00
P		*	ns				
WA (*n* = 38)							
*A*_n_	4	12	11	3	7	3	4
*A*_r_	2.675	3.50	4.42	4.42	4.42	4.42	2.63
*H*_o_	0.47	0.65	0.58	0.11	0.46	0.11	0.26
*H*_e_	0.62	0.71	0.86	0.10	0.74	0.29	0.61
P	ns	***	***	ns	**	***	***
coWA (*n* = 6)							
*A*_n_	2	2	6	1	3	2	1
*A*_r_	2.00	1.99	6.00	6.00	6.00	6.00	1.00
*H*_o_	0.67	0.40	1.00	0.00	0.83	0.33	0.00
*H*_e_	0.50	0.48	0.83	0.00	0.57	0.28	0.00
P	ns	ns	ns		ns	ns	
ceOR (*n* = 5)							
*A*_n_	3	2	4	2	2	2	3
*A*_r_	2.75	2.00	4.00	4.00	4.00	4.00	2.47
*H*_o_	1.00	0.67	1.00	0.20	1.00	0.40	0.60
*H*_e_	0.59	0.44	0.72	0.18	0.50	0.32	0.46
P	ns	ns	ns	ns	ns	ns	ns
VanIsl (*n* = 18)							
*A*_n_	1	3	7	1	4	2	3
*A*_r_	1.00	1.91	3.08	3.08	3.08	3.08	2.72
*H*_o_	0.00	0.36	0.47	0.00	0.20	0.11	0.19
*H*_e_	0.00	0.31	0.60	0.00	0.30	0.11	0.64
P	–	ns	ns	–	*	ns	***
Overall (*n* = 402)							
*A*_n_	9	16	15	6	8	10	6

Only populations with greater than five samples were used; *n *= number of samples used in genotyping and analyses; *A*_n_, number of alleles; *A*_r_, allelic richness; *H*_o_, observed and *H*_e_, expected heterozygosity; P, departures from Hardy–Weinberg equilibrium (–, not calculated, ns, not significant, *p <* *.05, **p <* *.01, ***p <* *.001. See Table 1 for population location abbreviations).

a Removed from subgroup clustering analyses due to missing data.

### 3.2 Mitochondrial DNA

We found 261 different haplotypes with overall haplotype diversity (*H*_d_) of 0.982, ranging from 0.707 (NNWBC) to 1.000 (11 populations; Table 1). Nucleotide diversity (π) ranged from 0.002 (VanIsl, coWA, NL, and NM) to 0.014 (NEWA; Table 1).

**Figure 3:**
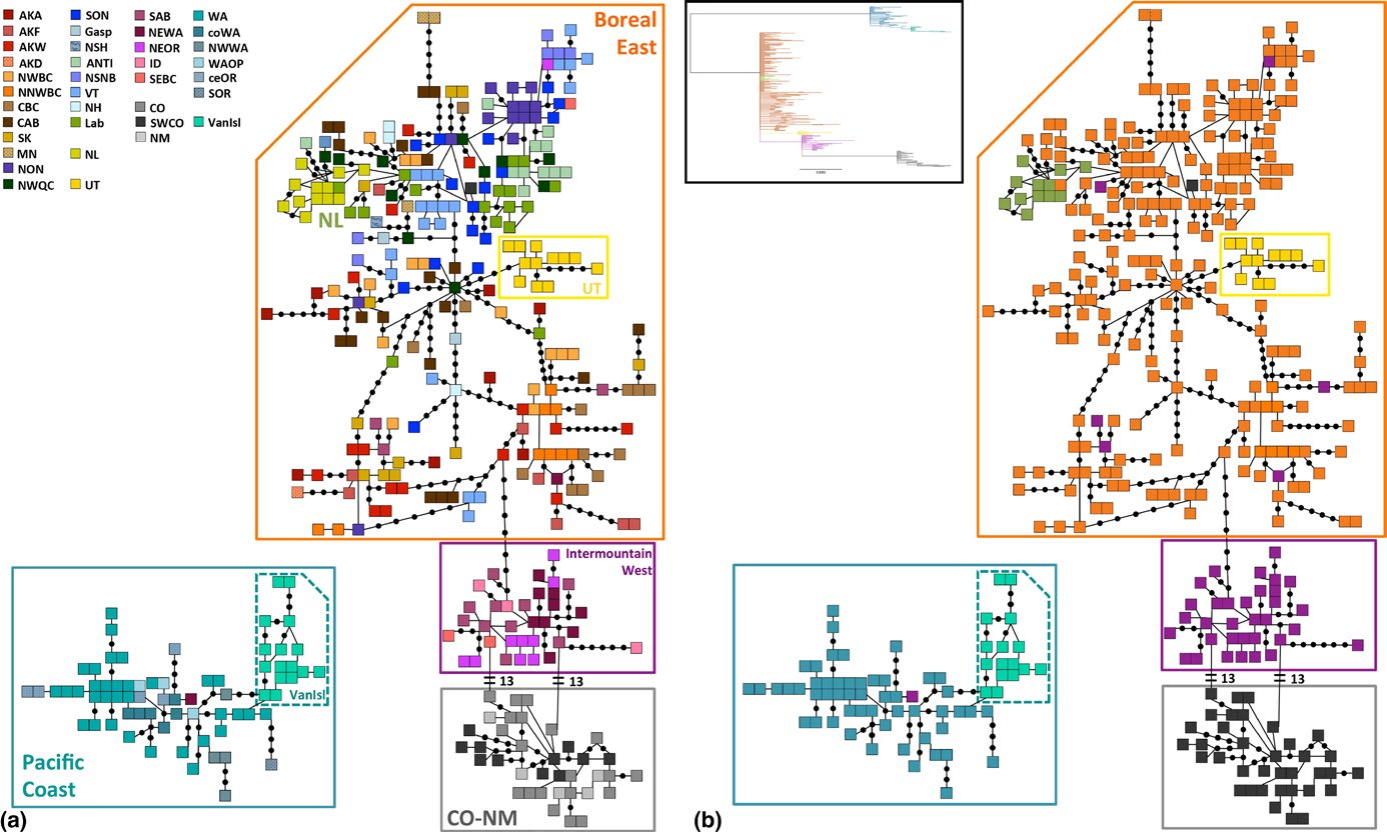
Statistical parsimony network of mtDNA haplotypes. Statistical parsimony network of 261 gray jay mitochondrial DNA haplotypes for 375 individuals reflecting main haplogroups. Each square represents one individual, individuals with the same haplotype are adjacent, and black dots represent an inferred haplotype. In (a) colors correspond to sampled populations (see legend in top left) and (b) colors correspond to general haplogroups or population source. Population abbreviations and locations are given in Table 1. Box: Simplified phylogenetic tree with colors corresponding to sampled populations as in b)

The statistical parsimony network (Figure 3) shows at least seven haplogroups throughout North America: Pacific Coast; VanIsl; Intermountain West; Colorado‐New Mexico; UT; Boreal‐east; and NL (Table 1). We excluded populations with less than four birds from further mtDNA analyses. In pairwise comparisons of the remaining 28 populations, 353 of 378 Φ_ST_ values were significant (B‐H corrected *p *<* *.047; Table 3; Table S4).

**Table 3:**
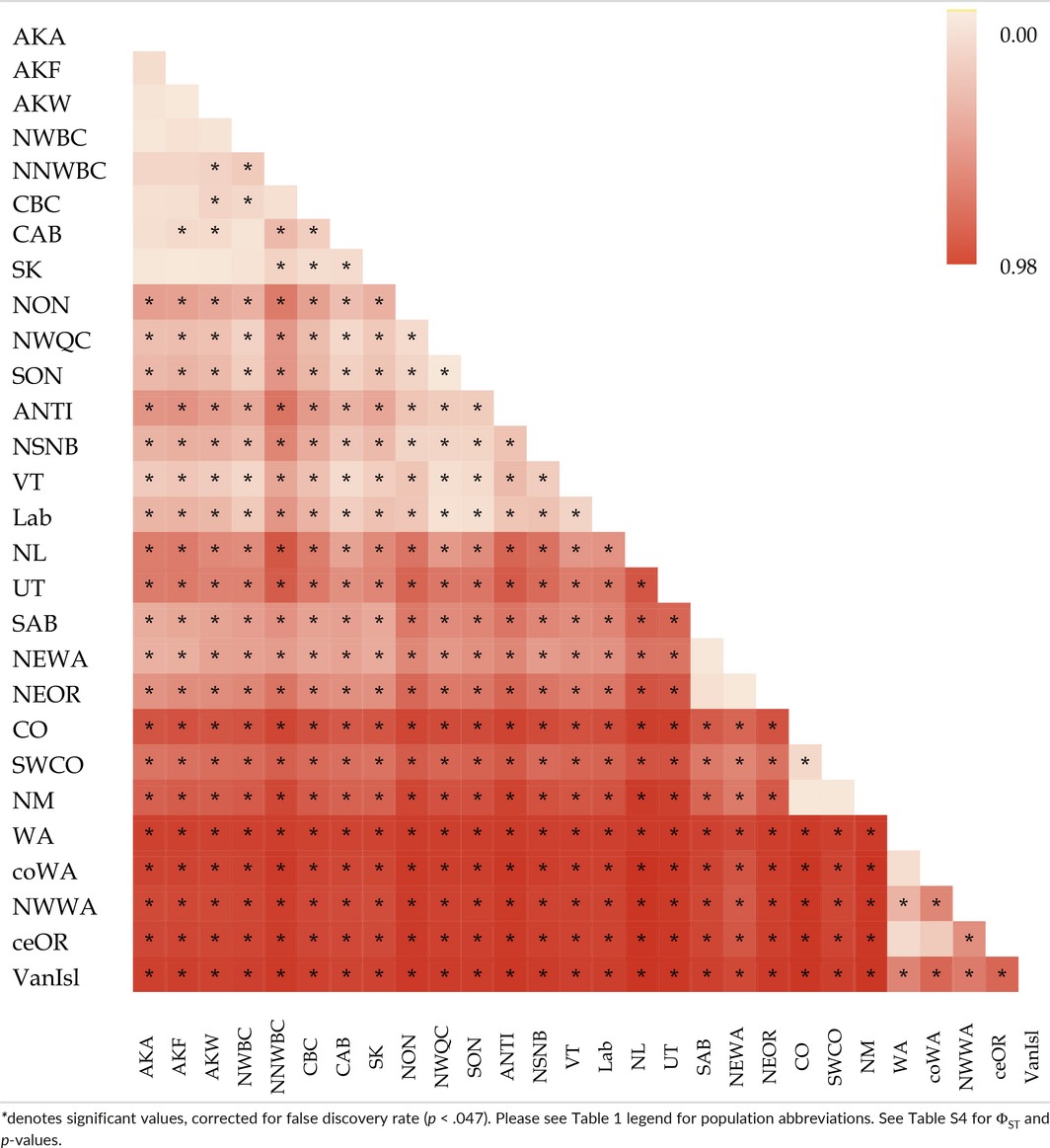
Heat map of pairwise Φ_ST_ values of population differentiation

A SAMOVA run with K = 7, accounted for the highest amount of variation among groups (79.57%, *F*_CT_ = 0.797, *p *<* *.0001; Table 4). SAMOVA population groupings corresponded with those suggested in the statistical parsimony network (Figure 3) and the same groups used in the analysis of molecular variance (AMOVA) to explain the most among group variation.

**Table 4:** Spatial analysis of molecular variance (SAMOVA) for gray jay mtDNA control region

	*df*	Variance component	% variation	Fixation index
Among groups	6	11.28	79.57	*F*_CT_ = 0.797**
Among populations, within groups	21	0.52	3.66	*F*_ST_ = 0.832**
Within populations	327	2.38	16.78	*F*_SC_ = 0.179**

The highest amount of between group variation was produced at K = 7. SAMOVA software assigned populations to seven groups that were identical to those found in the statistical parsimony network and assigned during AMOVA analysis. **denotes significance tests with *p *<* *.001. Group 1: AKA, AKF, AKW, NNWBC, NWBC, CBC, CAB, SK, NON, NWQC, SON, ANTI, VT, Lab, NSNB. Group 2: NL. Group 3: UT. Group 4: CO, SWCO, NM. Group 5: NEWA, NEOR, SAB. Group 6: WA, NWWA, coWA, ceOR. Group 7: VanIsl. Population abbreviations are explained in Table 1.

#### 3.2.1 Microsatellite DNA

A total of seven polymorphic microsatellite loci were used for analyses (Table S2). Twenty‐seven populations with five or more samples were included in general analyses and initial Bayesian analyses of population clustering. Total number of alleles for each locus ranged from six for MJG1 and ApCo41 to 16 in ApCo37 (Table 2). Overall allelic richness ranged from 1.86 for MJG1 to 4.4 for ApCo40, ApCo41, ApCo91, and Ck2A5A. Thirty‐eight of 189 loci‐population comparisons deviated significantly from Hardy–Weinberg equilibrium (Table 2).

**Figure 4:**
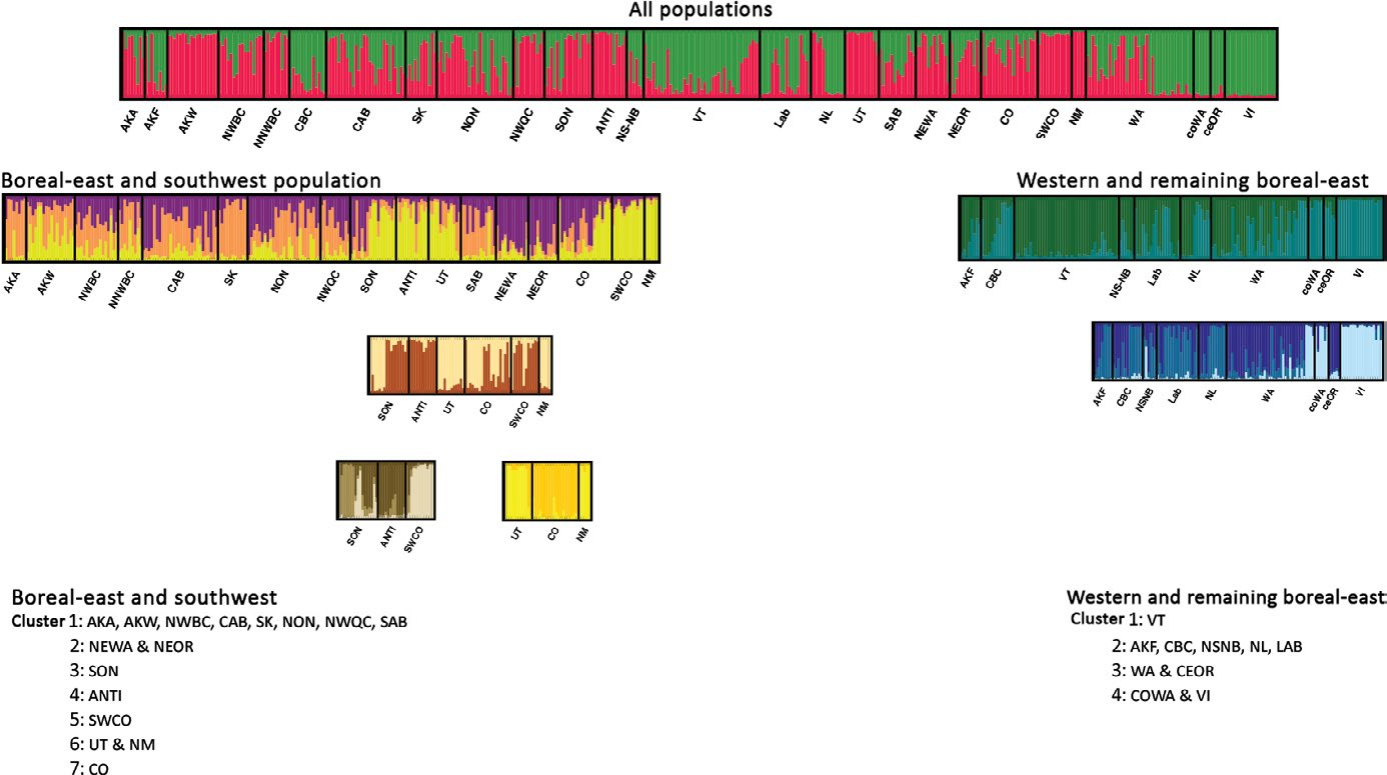
Bayesian clustering plots of gray jay microsatellite data

Significant differentiation was detected in 325 of 351 pairwise population comparisons (Table 5), with *F*_ST_ values ranging from 0.012 (*p *=* *.62) for NNWBC and AKW to 0.59 for NM and coWA (*p < *.001; Table S5). The initial structure clustering analysis suggested that the optimal number (*K*) of gray jay populations was two (mean LnP(*K*) = −5579.66; Δ*K* = 115.76; Figure 4). Further analysis of these two main groups indicates hierarchical structuring within each group. Among the first group, consisting of most Boreal‐east populations and populations in the intermountain west and southwestern US (CO, NM, SWCO, and UT), we detected seven distinct genetic clusters. The majority of Boreal‐east populations clustered into a single group, NEOR and NEWA clustered into a group, while, CO and SWCO clustered into single groups individually. UT and NM clustered into a single population, while ANTI and SON clustered together for the most part, although some individuals from SON clustered into a small separate group. The second cluster from our initial *K* = 2 analysis was composed of western and remaining boreal‐east populations. Again we found hierarchical structure, although there were fewer clusters within this region compared to the first main cluster. Within this second cluster, Vermont was a single cluster, the remaining boreal‐east populations (AKF, CBC, Lab, NSNB, and NL) clustered into a single cluster, while WA and ceOR clustered together, and coWA and VI clustered into a fourth group (Figure 4).

**Table 5:**
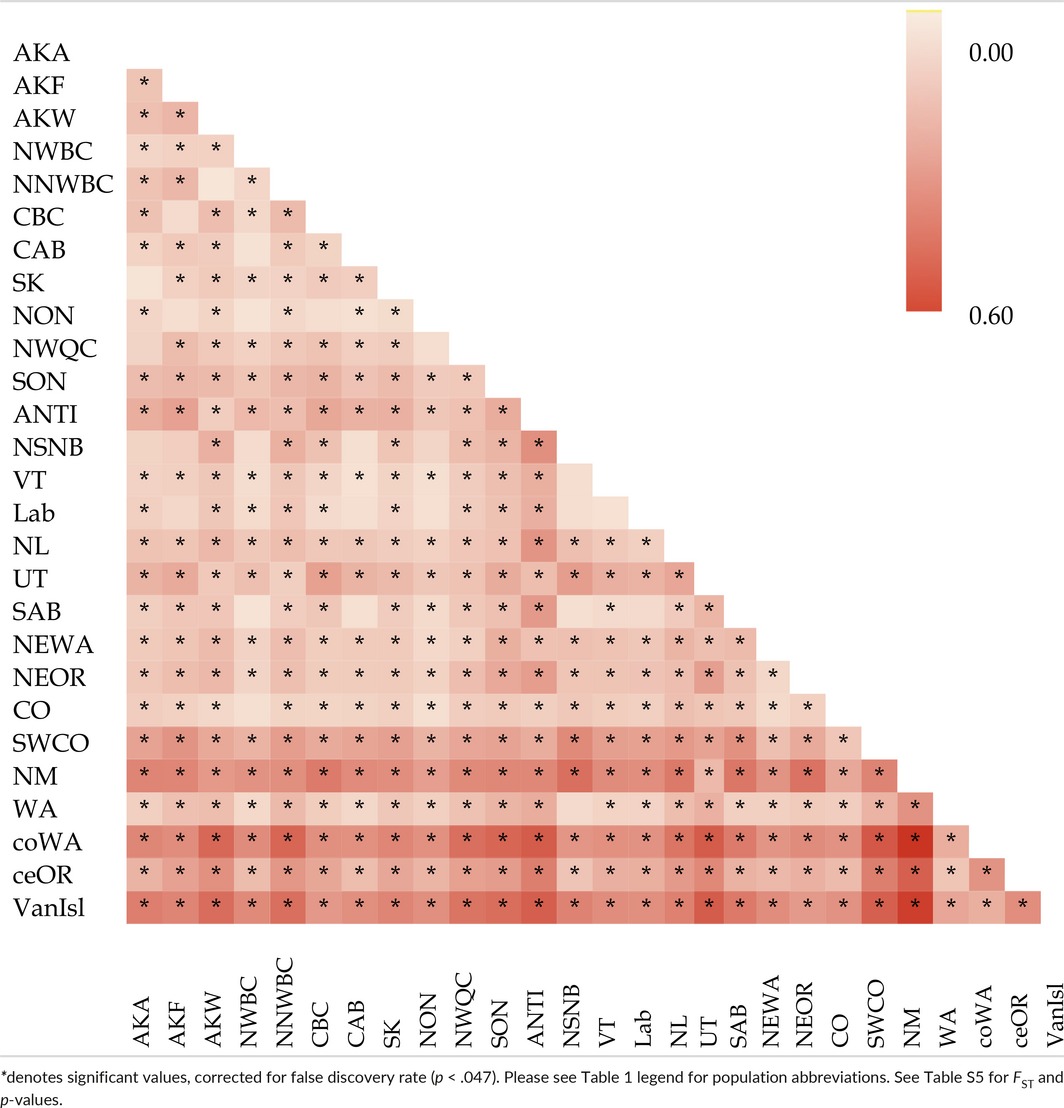
Heat map of pairwise *F*_ST_ values of population differentiation for seven microsatellite loci

### 3.3 Species distribution modeling

**Figure 5:**
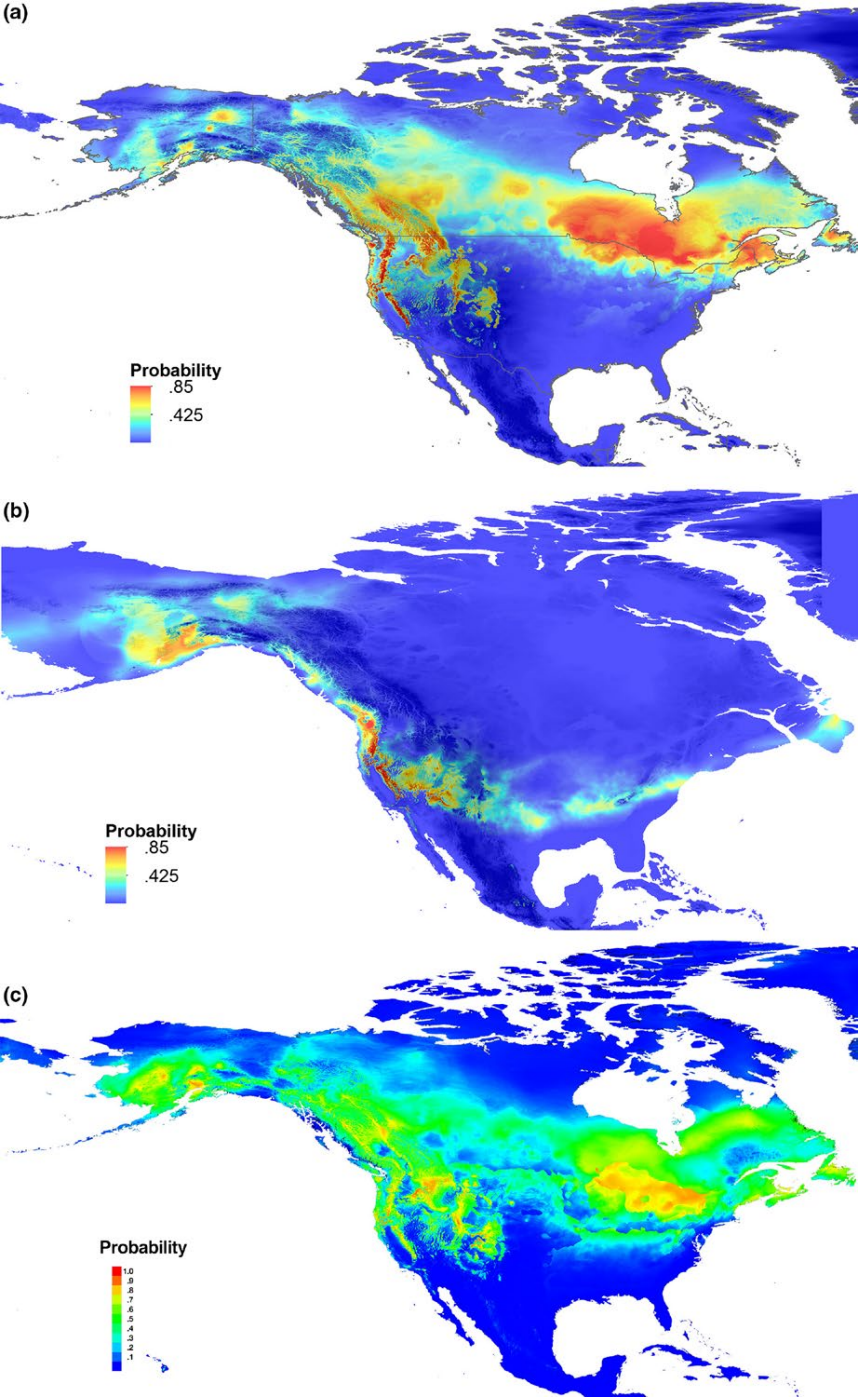
Predicted current and paleodistributions of gray jays in North America. (a) Current predicted range, (b) ~21 ka paleodistribution, and (c) ~120–140 ka (Last Interglacial) paleodistribution for gray jay in North America modeled using maxent software. Reds and oranges indicate increased probability of species occurrence; probability scale below, differing between C and A & B. Probability maps (a) and (b) are layered over digital elevation model (DEM). DEM legend is given in Figure 2

maxent modeling predicted a current range similar to that known for gray jays in North America with little variance (Figure 5a). Mean area under the curve (AUC) was 0.857 (*SD* = 0.012; training AUC range: 0.859–0.862, test AUC range: 0.842–0.870), suggesting that the models were reasonable as AUC values above 0.75 are considered “potentially useful” (Elith, 2000). Annual temperature (bio1; 33.2%), precipitation of coldest quarter (bio19; 29.8%), annual precipitation (bio12; 14.5%), and mean diurnal temperature range (bio2; 14.3%) were the largest contributors to the model contributing 91.8%, in addition to having the highest permutation importance (39.7, 25.8, 7.3, and 7.4, respectively) as supported by jackknifing.

When the model used current conditions to predict suitable gray jay habitat during the last glacial maximum (LGM), five main areas have a high probability of suitable gray jay habitat (0.5–0.8): most of Alaska and parts of Beringia, two areas in the southern Rockies, the SE US through Tennessee and Virginia, and the Pacific Coast including parts of Vancouver Island, Washington and Oregon (Figure 5b). The model also shows suitable gray jay habitat may have existed near Newfoundland. During the last interglacial period (LIG; ~120–140), suitable gray jay habitat reflected that of the present distribution, with greater levels of suitable habitat in the Intermountain West and southern Ontario and Quebec (Figure 5c). This suggests that gray jays expanded into previously occupied areas after the ice sheets of the LGM receded (Figure 5c).

### 3.4 Barrier analyses

**Figure 6:**
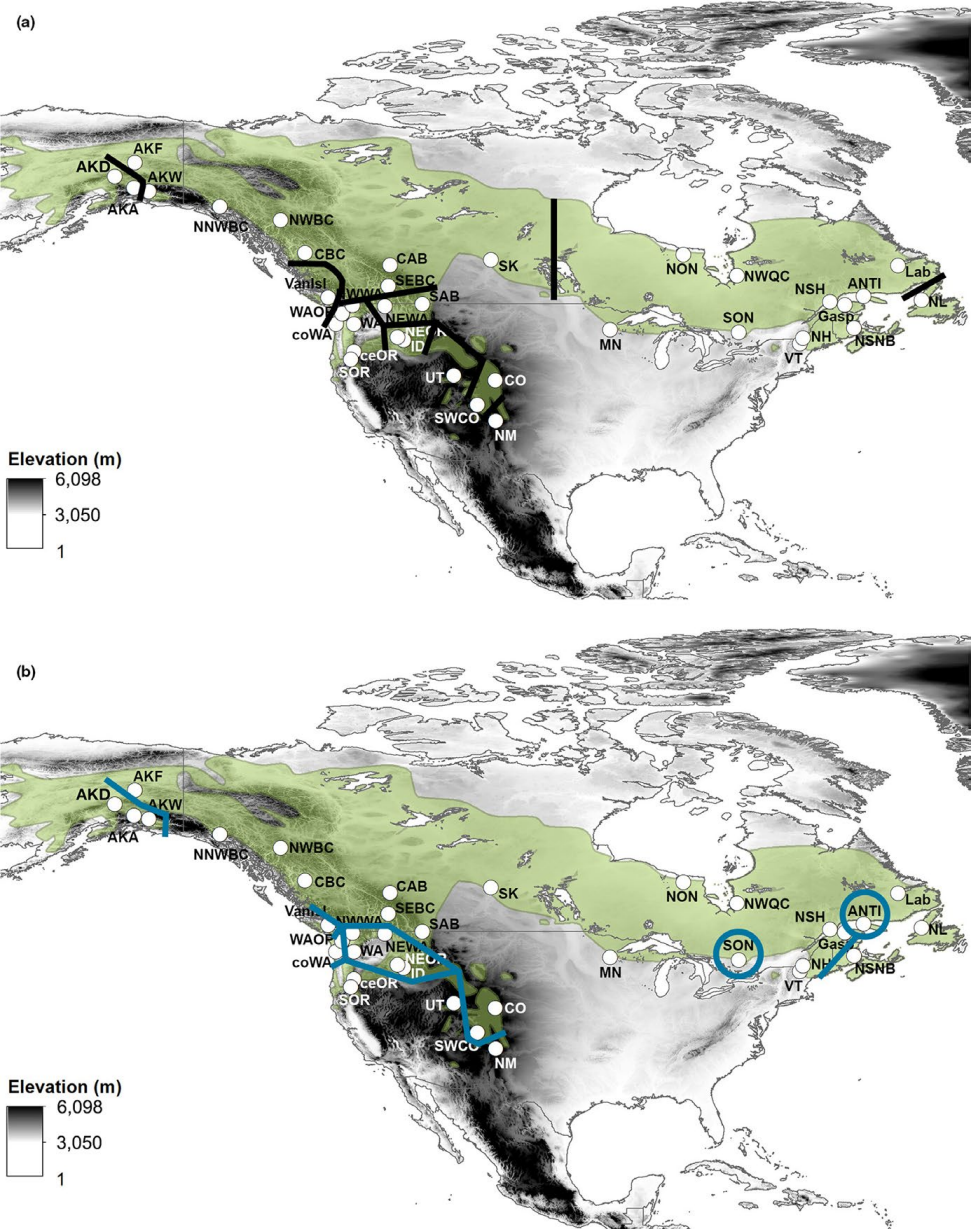
Analyses of barriers to gene flow for (a) mtDNA and (b) microsatellite markers

Using BARRIER, we found congruent patterns between mtDNA and microsatellite markers (Figure 6). The majority of barriers identified were located in the western portion of the gray jay range and appear to correspond with the location of mountain ranges, water barriers, or breaks in suitable habitat. While patterns were mostly congruent between marker sets, there were some differences. In particular, mtDNA identified a barrier between Newfoundland and mainland populations, but microsatellite patterns did not detect any potential barriers in this region. Additionally, both Vermont (VT) and Southern Ontario (SON) appear to be separated from all other nearby populations based on microsatellite patterns, whereas our analysis with mtDNA detected no barriers between VT and SON and other nearby populations. Overall, barrier locations are congruent with mtDNA and microsatellite cluster analysis results (SAMOVA and STRUCTURE).

Our dbRDA models at the individual level found a significant relationship between the six environment variables we examined and both mtDNA and microsatellite genetic structure (Table 6). Similar environmental variables appear to influence both mtDNA and microsatellite genetic structure, although environment accounted for greater variance with respect to mtDNA genetic structure than microsatellite genetic structure. Precipitation during the coldest quarter accounted for twice as much variance (*r* ^2^ = .29) than geographic distance (*r* ^2^ = .14) or geographic location (*r* ^2^ = .13), while glaciation, altitude, and mean temperature were all significant, but accounted for a relatively small portion of the variance. For microsatellite genetic structure, the six variables accounted for a very small portion of variance (0.01–0.02). Similar to mtDNA patterns, precipitation during the coldest quarter was the top predictor of genetic variation among the six we tested (*F* = 17.11, *p *=* *.001). Our results indicate a weak effect of isolation by distance on genetic patterns overall, further suggesting the influence of barriers on genetic structure in gray jays.

**Table 6:** dbRDA model results

	mtDNA		microsatellite	
	%Var	*p*	%Var	*p*
Latitude and longitude	0.13	.001	0.02	.001
Geographic distance	0.14	.001	0.02	.001
Mean annual temperature	0.04	.001	0.01	.001
Precipitation during coldest quarter	0.29	.001	0.02	.001
Altitude	0.06	.001	0.01	.001
Glaciation	0.10	.001	0.02	.001

%Var shows the percentage of genetic variation for mtDNA and microsatellite patterns explained by each of the biotic and abiotic variables tested in our dbRDA models.

## 4 DISCUSSION

Geographic structuring and population differentiation suggest different evolutionary histories for gray jays in North America. Gray jays are partitioned into seven geographically distinct mitochondrial groups throughout their range: Pacific Coast; Vancouver Island; Intermountain West; CO‐NM; Utah; Newfoundland; and Boreal‐east. Microsatellite markers support similar breaks with significant differentiation (*F*_ST_) between most populations and clustering roughly corresponding to larger mitochondrial haplogroups. Exceptions to this include some splits amongst Borealeast populations, inclusion of AKF and CBC with Pacific Coast groups, and several populations that were difficult to consistently assign to a single cluster, suggesting nuclear genetic admixture between some groups.

### 4.1 LGM refugia and patterns of postglacial colonization

High‐mitochondrial genetic diversity exists within most groups, suggesting few founder events occurred during gray jay recolonization after deglaciation. Most areas have haplotype diversity approaching one. High‐haplotype diversity and few shared haplotypes between populations also suggest limited maternal gene flow among groups, as might be expected in a sedentary species (Barrowclough et al., 2004; Bertrand et al., 2014; Burg et al., 2006; Graham & Burg, 2012).

Mitochondrial DNA patterns in the gray jay suggest long‐term isolation in multiple refugia and low levels of gene flow following the retreat of the ice sheets. Species distribution modeling (SDM) and fossil data (Wetmore, 1962) reinforce the presence of multiple southern refugia and SDM data support a northern refugium. While SDM shows refugia during the LGM and these maintained isolation of genetically distinct groups (e.g., CO‐NM, UT), isolation during earlier glaciations likely created many of the haplogroups seen. In addition, SDM modeling for the LIG suggests a similar distribution to that at present, though with greater concentration of suitable habitats in areas near refugia, corresponding to mtDNA groups.

While our results are similar to the genetic patterns shown by van Els et al. (2012), our increased sampling indicates greater population structuring than that found in the previous study. For example, Φ_ST_ and SAMOVA results based on mtDNA indicate individuals on Vancouver Island were likely isolated in a different refugium from those on the mainland as evident from the distinct sets of haplotypes on Vancouver Island. The Pacific Coast populations have remained relatively isolated from other populations, and SDM shows suitable habitat both on the mainland and Vancouver Island during the LGM and LIG. Other North American taxa show evidence of isolation on the mainland (Barrowclough et al., 2004; Carstens, Brunsfeld, Demboski, Good, & Sullivan, 2005; Godbout, Fazekas, Newton, Yeh, & Bousquet, 2008; Graham & Burg, 2012), and a few on Vancouver Island, possibly in ice‐free portions of the Brooks Peninsula on northern Vancouver Island during the LGM (Godbout et al., 2008; Walser, Holderegger, Gugerli, Hoebee, & Scheidegger, 2005).

Further, our increased sampling indicates that populations in southern British Columbia and Alberta were colonized from a shared refugium east of the Cascades. Gray jay populations in the IMW group contain high levels of genetic diversity and are genetically isolated from adjacent populations, a pattern suggestive of long‐term isolation. The Clearwater refugium has been suggested as a refugium for other species in the area (Godbout et al., 2008; Shafer et al., 2010), including emerging pollen evidence for *Picea* species (Herring & Gavin, 2015). While our mtDNA data support isolation, the paleodistribution modeling data do not show evidence of suitable gray jay habitat in the area 21 kya, though highly suitable habitat likely existed in this area during the LIG. Alternatively, the IMW group may have survived the LGM in a refugium slightly farther south than the Clearwater refugium; paleodistribution models suggest that suitable habitat for gray jays existed in northern Nevada.

Our remaining haplogroups coincide with those patterns observed by van Els et al. (2012). These patterns indicate the potential for at least four other refugia during the LGM. Populations in CO‐NM likely persisted in a single refugium, while UT populations were isolated in a separate refugia. The Boreal‐east group contains a large number of diverse haplotypes spread over large geographic areas with most populations containing high haplotype and nucleotide diversity. One exception is the NL population. Reduced genetic diversity and a clustered set of haplotypes in NL gray jays could be the result of a founder effect or a population bottleneck and no gene flow due to the Strait of Belle Isle acting as a dispersal barrier as it does in other species (Kyle & Strobeck, 2003; Lait & Burg, 2013), although SDM suggests the presence of an Atlantic refugium near Newfoundland and such a refugium is supported by a number of species (Boulet & Gibbs, 2006; Jaramillo‐Correa et al., 2009; Lait & Burg, 2013).

With respect to the remaining populations in the Boreal‐East, areas in the SE US and Beringia could have supported populations of gray jays during the LGM based on suitable habitat models. Fossil evidence shows gray jays were in Tennessee and Virginia during the LGM, (Wetmore, 1962), though populations are no longer present in those areas. Many other high latitude species survived the LGM in the eastern US (Jaramillo‐Correa et al., 2009; Graham & Burg, 2012; (Gérardi, Jaramillo‐Correa, Beaulieu, & Bousquet, 2010). Contemporary samples from Alaska, near the Beringia refugium, include haplotypes scattered throughout the statistical parsimony network lending support to a Beringia refugium for gray jays. Alternatively, this could suggest a diverse number of founders from other populations colonizing Beringia after deglaciation. However, given known geographical patterns of deglaciation, genetic evidence from other species (Lait & Burg, 2013; Shafer, Côté, & Coltman, 2011; Zink & Dittmann, 1993), and the diverse nature of haplotypes in Alaska, the former scenario is more likely.

### 4.2 Tree refugia

Gray jays are dependent on forested habitat and, in particular, several species of spruce trees (*Picea* spp.). CO‐NM, UT, and IMW groups are all closely associated with Engelmann and blue spruce, which are highly fragmented in the southern portion of their range (i.e., UT and CO; Ledig, Hodgskiss, & Johnson, 2006). Populations of Engelmann and blue spruce in the IMW and NE UT are genetically distinct (cpDNA) and physically isolated from each other by the Snake River Basin (Ledig et al., 2006), corresponding to the mitochondrial DNA patterns found here.

Further support for gray jay colonization throughout the Boreal‐East from both a Beringia and a southeastern refugium comes from phylogeographic studies of spruce (*Picea* spp; Jaramillo‐Correa et al., 2009). The strong association of gray jays with spruce species in these areas (Strickland & Ouellet, 2011) means it is possible that the birds may have followed the colonization of spruce into previously glaciated areas, a pattern seen in other boreal species (Burg et al., 2006; Graham & Burg, 2012). The colonization by spruce is suggested to have occurred from multiple refugia north (Beringia) and south (both east and west of the Appalachian Mountains), particularly for white spruce (*Picea glauca*; Jaramillo‐Correa et al., 2009; de Lafontaine, Turgeon, & Payette, 2010). Black spruce (*Picea mariana*) has a similar colonization history in the east. However, west of the Rocky Mountains, black spruce is thought to have colonized only from a southern, Pacific refugium (Gérardi et al., 2010), contrary to the pattern of colonization from multiple refugia that we suggest for gray jays in mainland British Columbia.

### 4.3 Dispersal barriers and peripheral isolation

Congruent patterns between mtDNA and microsatellite markers suggest that similar factors are influencing historical and contemporary genetic patterns. We found limited support to suggest that distance or environmental factors are influencing genetic patterns, in this species, as has been shown in other North American resident species (Graham & Burg, 2012; Lait, Friesen, Gaston, & Burg, 2012). Precipitation during the coldest quarter explained a high portion of variance, but this likely reflects how similar the majority of populations in the boreal‐east are. Instead other dispersal barriers appear to restrict gene flow in gray jays. Barriers include large bodies of water (Strait of Belle Isle and the Salish Sea), large areas of unsuitable habitat (Columbia, Wyoming, and Great Basins) and, in some areas, possibly mountains (Columbia Mountains in southeast BC), similar to patterns in other North American species (Adams & Burg, 2015; Klicka, Spellman, Winker, Chua, & Smith, 2011; Manthey, Klicka, & Spellman, 2011). With the exception of nine individuals, no haplotypes are shared between the mitochondrial haplogroups suggesting limited female movement. Given that both mtDNA and microsatellite markers show similar levels of genetic structure, these results suggest limited male and female movement across landscapes.

Water barriers appear to influence genetic structure, as we observed significant genetic differences (based on both Φ_ST_ and *F*_ST_ values) between mainland populations and the three island populations we sampled: Vancouver Island, Anticosti Island, and Newfoundland. Additionally, haplotype analyses and cluster analyses indicate genetic isolation of all three islands, although Anticosti groups with mainland populations based on haplotype analysis, while clustering analysis did not distinguish Newfoundland from other mainland populations. Similar patterns of genetic isolation for both plant and animal species have been found for Vancouver Island and Newfoundland, though usually with high‐resolution nuclear markers and not organellar DNA. The Salish Sea restricts populations on Vancouver Island (e.g., Steller's jay (*Cyanocitta stelleri*; Burg et al., 2005), chestnut‐backed chickadee (*Poecile rufescens*; Burg et al., 2005)), and the Strait of Belle Isle isolates populations on Newfoundland (e.g., pine marten (*Martes americana*; Kyle & Strobeck, 2003); boreal chickadee (*P. hudsonicus*; Lait & Burg, 2013). Our work supports these two water bodies as barriers to dispersal and suggests that the Gulf of Saint Lawrence also acts as a barrier to dispersal.

Though close in proximity to each other (~530 km apart), the northern Colorado and Utah populations are highly differentiated for both mitochondrial and nuclear DNA. Two possible reasons are large areas of unsuitable habitat or isolation of peripheral, disjunct populations. The Great Basin to the northwest, Wyoming Basin to the north/northeast and Snake River Basin to the north/northwest all act as barriers to dispersal and gene flow with neighboring populations. The divergence between Colorado and neighboring populations in Utah, but not between Colorado and neighboring populations in New Mexico, has been observed in other taxa (Albach, Schonswetter, & Tribsch, 2006; Runck & Cook, 2005). Most notably, congruent patterns of isolation are found in Engelmann and blue spruce (Ledig et al., 2006), which were restricted to higher elevations and isolated on mountains as aridification occurred in the Great and Wyoming Basins. In addition, both the UT and CO populations are currently ~390–700 km, respectively, to the nearest population within the contiguous portion of the gray jay range. Peripheral isolation may also explain the high differentiation and isolation in these disjunct populations. In other taxa, peripheral populations are more likely to be isolated due to reduced gene flow, which is particularly pronounced for disjunct populations (Burg et al., 2006; Eckert, Samis, & Lougheed, 2008). East‐central Arizona populations may show similar patterns of isolation based on their proximity to and clustering as a subspecies with other groups in this area (Strickland & Ouellet, 2011); we did not collect any samples from the subpopulation to confirm this pattern.

The Intermountain West (NEWA, SAB, NEOR, SEBC, and ID) group, unlike some of the other isolated populations, occupies a central portion of the gray jay range, yet they are genetically distinct from surrounding groups for both mitochondrial and nuclear markers. Birds in this area are isolated from adjacent populations by the Columbia Basin/Okanogan Highlands to the west (Pacific populations), Columbia Mountains and Rocky Mountain Trench to the north and Columbia Mountains to the east (Boreal‐east), and the Snake River Basin to the south (Colorado and Utah). A similar genetic break occurs in mtDNA patterns in Engelmann spruce (Ledig et al., 2006) and Douglas fir (Gugger, Sugita, & Cavender‐Bares, 2010); both species of trees that gray jays are closely associated with in the Intermountain West area (Strickland & Ouellet, 2011).

### 4.4 Marker choice and overall patterns

While some studies question using a highly variable marker like control region versus ND2 or cytochrome b for phylogeographic and phylogenetic studies, previous work has shown that this marker can be used to resolve deep splits in evolutionary history among avian species (Barker, Benesh, Vandergon, & Lanyon, 2012) and of corvids in particular (Saunders & Edwards, 2000). Within a single species, some loci may not be variable enough to detect differences between populations (e.g., cytochrome b (Steeves, Anderson, McNally, Kim, & Friesen, 2003) versus control region (Steeves, Anderson, & Friesen, 2005) in masked boobies (*Sula dactylatra*)). Thus, using control region sequences in this study provides a valuable comparison and complement to previous research.

Similar haplogroup patterns are found in van Els et al. (2012); however, our work differs in several ways. We suggest that gray jays fall into seven haplogroups across North America compared to four; additional groups are Utah, which is similar to the Boreal group as in van Els et al. (2012) but with higher resolution control region data create a distinct group, and Vancouver Island, with higher diversity in the CO‐NM and Pacific Coast groups. While some evidence exists in our paleodistribution model for a Newfoundland LGM refugium, also suggested by van Els et al. (2012), genetic data in both studies do not support this refugium and rather suggest a case of long‐term isolation, possibly in a nearby refugium. One benefit to using the control region is that it allows us to distinguish additional genetic splits (e.g., NL) that might not be as evident using less variable markers. Adding microsatellite markers to our analyses provided additional support and resolution for geographic patterns. Strong differentiation between most populations is similar to that found with mitochondrial DNA, and clustering provides additional insights into patterns throughout the range. Though van Els et al. (2012) suggest that three distinct morphogroups exist, similar to that found in Sibley (2000), our observations of morphology and plumage in the field suggested less distinct groups with greater clinal variation. One notable exception is that of birds in Newfoundland, which were heavier and had shorter tarsi than other groups (Dohms, 2016). Overall, we did not observe distinct differences corresponding to haplogroups in our work.

### 4.5 Conclusions and future research

Gray jay populations are highly differentiated, likely a consequence of limited dispersal for both males and females. Historical and contemporary gene flow is influenced by glaciation, barriers to movement such as large bodies of water and large areas of unsuitable habitat, and peripheral isolation. Additional research could include greater numbers of microsatellite loci or other nuclear markers to further enhance and complete our understanding of gray jay history and contemporary gene flow in North America.

Overall our findings provide greater insight into the ecology, evolution, and conservation of boreal organisms. For example, gray jay geographic genetic patterns are similar to those found in spruce species, the conifer genus most commonly associated with preferred gray jay habitats, suggesting a close association between habitat and diversification in this species. Given this parallel, we would recommend future comparative phylogeography research that integrates genetic markers and species distribution modeling for gray jay, spruce, and other codistributed species. Incorporating this integrative approach is important, given that boreal habitats are under threat, as a result of climate change.

## CONFLICT OF INTEREST

None declared.

## AUTHORS CONTRIBUTIONS

KMD designed study, collected data, conducted laboratory work, analyzed and interpreted results, wrote and edited manuscript, and approved submitted manuscript. BAG collected data, analyzed and interpreted results, wrote and edited manuscript, and approved submitted manuscript. TMB designed study, interpreted results, edited manuscript, provided research facilities and funding, and approved submitted manuscript.
